# Cerebral Metabolic Changes During Visuomotor Adaptation Assessed Using Quantitative fMRI

**DOI:** 10.3389/fphys.2020.00428

**Published:** 2020-05-08

**Authors:** Catherine Foster, Jessica J. Steventon, Daniel Helme, Valentina Tomassini, Richard G. Wise

**Affiliations:** ^1^Cardiff University Brain Research Imaging Centre (CUBRIC), School of Psychology, Cardiff University, Cardiff, United Kingdom; ^2^Cardiff University Brain Research Imaging Centre (CUBRIC), School of Physics and Astronomy, Cardiff University, Cardiff, United Kingdom; ^3^Neuroscience and Mental Health Research Institute (NMHRI), School of Medicine, Cardiff University, Cardiff, United Kingdom; ^4^Department of Anaesthetics and Intensive Care Medicine, Cardiff University School of Medicine, Cardiff, United Kingdom; ^5^Division of Psychological Medicine and Clinical Neurosciences, School of Medicine, Cardiff University, Cardiff, United Kingdom; ^6^Department of Neuroscience, Imaging and Clinical Sciences, “G. D’Annunzio University” of Chieti-Pescara, Chieti, Italy; ^7^Institute for Advanced Biomedical Technologies (ITAB), “G. D’Annunzio University” of Chieti-Pescara, Chieti, Italy

**Keywords:** calibrated fMRI, cerebral blood flow, functional MRI, motor adaptation, oxygen metabolism

## Abstract

The brain retains a lifelong ability to adapt through learning and in response to injury or disease-related damage, a process known as functional neuroplasticity. The neural energetics underlying functional brain plasticity have not been thoroughly investigated experimentally in the healthy human brain. A better understanding of the blood flow and metabolic changes that accompany motor skill acquisition, and which facilitate plasticity, is needed before subsequent translation to treatment interventions for recovery of function in disease. The aim of the current study was to characterize cerebral blood flow (CBF) and oxygen consumption (relative CMRO_2_) responses, using calibrated fMRI conducted in 20 healthy participants, during performance of a serial reaction time task which induces rapid motor adaptation. Regions of interest (ROIs) were defined from areas showing task-induced BOLD and CBF responses that decreased over time. BOLD, CBF and relative CMRO_2_ responses were calculated for each block of the task. Motor and somatosensory cortices and the cerebellum showed statistically significant positive responses to the task compared to baseline, but with decreasing amplitudes of BOLD, CBF, and CMRO_2_ response as the task progressed. In the cerebellum, there was a sustained positive BOLD response in the absence of a significant CMRO_2_ increase from baseline, for all but the first task blocks. This suggests that the brain may continue to elevate the supply energy even after CMRO_2_ has returned to near baseline levels. Relying on BOLD fMRI data alone in studies of plasticity may not reveal the nature of underlying metabolic responses and their changes over time. Calibrated fMRI approaches may offer a more complete picture of the energetic changes supporting plasticity and learning.

## Introduction

The brain retains a lifelong ability to adapt through learning and in response to injury or disease-related damage, a process known as functional neuroplasticity. Residual neuroplasticity in chronic diseases such as Multiple Sclerosis (MS), or following stroke, can be harnessed in rehabilitation strategies to promote recovery of function. However, the neuronal and vascular mechanisms underlying plasticity are not fully understood. Adequate energy delivery in the form of cerebral blood flow (CBF), which carries oxygen, glucose and other nutrients to tissue, is essential for healthy neuronal function, as is the capacity to metabolize these substrates. In MS for example, there is evidence of both CBF (D’haeseleer et al., 2011; Ota et al., 2013) and metabolic dysfunction (Kidd et al., 1999; Ge et al., 2012; Fan et al., 2015) which may play a central role in limiting plasticity. The neural energetics underlying functional brain plasticity have not been thoroughly investigated experimentally in the healthy human brain. A better understanding of the blood flow and metabolism changes which occur during motor skill acquisition, and which facilitate plasticity, is needed before characterization in disease, and subsequent translation to inform treatment interventions to maintain or recover function.

Calibrated fMRI enables measurement of regional CBF and relative changes in the rate of cerebral metabolic rate of oxygen consumption (CMRO_2_) through the addition of hypercapnic calibration (Davis et al., 1998; Hoge et al., 1999) during dual-acquisition of BOLD and CBF weighted images. The technique has potential applications in identifying clinically relevant abnormalities in vascular and metabolic function which may not be evident using BOLD fMRI alone. CBF and CMRO_2_ provide additional information which aids interpretation of fMRI studies of aging and disease where neurovascular coupling (NVC) is likely to be altered (Restom et al., 2008). For example, greater BOLD responses with increasing age during a Stroop task have been reported, alongside a reduced CMRO_2_ increase in response to the task (Mohtasib et al., 2012). This suggests that as CBF was unaffected by age in this cohort, the greater BOLD signal changes were due to a reduction in the CMRO_2_ response. As CMRO_2_ and neuronal firing are closely coupled (Mathiesen et al., 1998; Martindale et al., 2003), a decreased neural response with age is a possible explanation for these results. Such changes in vascular reserve and NVC would not have been revealed by BOLD fMRI alone, and the results demonstrate the value of calibrated fMRI in studies where cerebral energetics may be altered by experimental conditions or over time.

**FIGURE 1:**
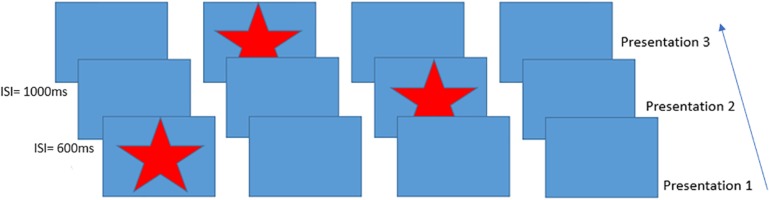
Schematic of the Serial Reaction Time task presentation, inter-stimulus intervals on the left indicate example times between trials. This figure shows an example of the presentation of the first three items of the 12-item sequence. Participants responded to each star position using a handheld 4-button response box.

The aim of the current study was to measure BOLD, CBF and CMRO_2_ responses during performance of a serial reaction time (SRT) task (Nissen and Bullemer, 1987) using calibrated fMRI. In the SRT task, participants respond to a sequence of stimuli that appear one-by-one at various locations on a screen. Participants respond by indicating the current stimulus location which follows a repeating pattern (see Figure 1 which depicts the first three presentations in the sequence) allowing participants to identify this sequence with practice, improving the accuracy and speed of responses. Visuomotor task performance can be improved over short periods of time, accompanied by hemodynamic changes in task-relevant regions, which are thought to reflect short-term plasticity in the adult brain (Olson et al., 2006; Fernández-Seara et al., 2009; Shannon et al., 2016). Previous work has demonstrated BOLD and CBF task responses in motor and visual cortex as well as prefrontal regions and the cerebellum during task performance (Ungerleider et al., 2002). BOLD signal reductions over time, due to task adaptation have also been observed within a single MRI session (Floyer-Lea and Matthews, 2004; Shannon et al., 2016). Therefore, we expected to observe reduced BOLD signal responses in task-relevant areas as motor adaptation occurred. CBF and CMRO_2_ may change dynamically with adaptation to the task in ways that are not visible by looking solely at the BOLD response, as it is the result of changes in vascular and metabolic processes. For example, although BOLD and CBF responses have been reported during motor skill learning task (Olson et al., 2006), it is not clearly established whether their changes over time follow similar patterns or whether there are also alterations in the CMRO_2_ response, as previous works have reported both no changes (Madsen et al., 1995) and reductions in CMRO_2_ response (Shannon et al., 2016) during skill learning.

To examine motor task adaptation, BOLD, CBF, and CMRO_2_ responses were calculated for each block of an SRT task in regions which showed reducing BOLD and CBF responses across the task. Changes in each parameter from baseline were investigated along with differences between brain regions and task blocks. Lastly, regression analysis was conducted to determine whether CBF, CMRO_2_ or BOLD predicted task performance.

## Materials and Methods

### Participants

A total of 20 right-handed, healthy participants (10 females, mean age 25 ± 4.6) took part in this study. All participants were non-smokers and educated to university level. The study was approved by the Cardiff University School of Psychology Research Ethics Committee and performed in accordance with the guidelines stated in the Cardiff University Research Framework (version 4.0, 2010). Informed written consent was obtained for all subjects.

### Imaging

Imaging was performed on a whole body 3T MRI (GE Excite HDx, Milwaukee WI, United States) system using an 8-channel receive-only head coil. Simultaneous perfusion and BOLD weighted data were acquired with a PICORE QUIPSS II (Wong et al., 1998) pulsed arterial spin labelling (PASL) sequence (non-commercial) with a dual-echo gradient-echo readout (Liu et al., 2002) and spiral k-space acquisition (Glover, 1999).

Imaging parameters for functional scans (task and hypercapnic calibration) were: TR = 2.4 s, TE1 = 2.7 ms, TE2 = 29 ms, TI1 = 700 ms, TI2 = 1.5 s (most proximal slice), FOV = 19.8 cm, flip angle = 90° matrix size = 64 × 64, slice thickness 7 mm, 1.5 mm gap, 3.1 mm in plane resolution with 15 slices. Label thickness was 200 mm with a 10 mm gap between the end of the label and the most proximal imaging slice. A separate single volume M_0_ scan was acquired using the same parameters, except TR = 4 s, to measure the equilibrium brain tissue magnetization of cerebrospinal fluid (CSF) for absolute CBF estimation. For registration, a 3D T1-weighted fast spoiled gradient echo sequence was acquired; TR = 7.9 ms, TE = 3 ms, 256 × 256, slice thickness = 1 mm, giving a resolution of 1 mm^3^.

Physiological monitoring was performed using a respiratory belt placed just below the ribs to monitor ventilation and a pulse oximeter to obtain cardiac traces. A sampling line connected to a tightly fitted face mask (Quadralite Intersurgical, Wokingham, Berkshire, United Kingdom) was used to record expired P_*ET*_CO_2_ and P_*ET*_O_2_ concentrations using the Biopac system (Biopac^®^, Worcestershire, United Kingdom). The face mask was connected to a breathing circuit used to deliver gas mixtures and followed the design of Tancredi et al. (2014). The MEDRAD system (MEDRAD, Pittsburgh, PA, United States) was used to monitor blood arterial O_2_ saturation during hypercapnia.

### Visuomotor Task

The SRT (Nissen and Bullemer, 1987) is a visuomotor task which has been used previously in fMRI studies in healthy subjects as well as in patient groups such as MS, chronic stroke and Huntington’s Disease (HD) (Knopman and Nissen, 1991; Boyd and Winstein, 2001; Bonzano et al., 2011).

A modified version of the SRT developed by Nissen and Bullemer (1987) was used as the visuo-motor learning task during imaging acquisition. The task was projected via a screen inside the scanner at a frame rate of 60 Hz and a resolution of 1024 × 768. A star appeared on the screen in a sequence of four boxes (Figure 1), participants responded by pressing the corresponding button on a button box in their right hand. The 12-minute task consisted of 6 blocks of a 12-item sequence repeated 6 times with variable inter-stimulus interval (600–1000 ms) interspersed with a pseudorandom sequence on every third block to assess response latency decreases related to task familiarization rather than sequence learning. Participants were not informed that there was a repeating sequence during task instructions. Sequence blocks are referred to as blocks S1–S6 throughout the text, and random blocks are referred to as blocks R1–R3.

### Hypercapnic Calibration

The SRT task was followed by hypercapnic calibration to obtain a measure of cerebrovascular reactivity (CVR) to CO_2_ for estimation of CMRO_2,_ using the fixed-inspired gas method. Participants breathed through a tight-fitting facemask as described above, gases were administered from gas cylinders connected to an in-house built manually controlled flow meter system. Gases were piped through a mixing chamber with three feeding lines coming in for the delivery of medical air, 5% CO_2_, and medical oxygen. Medical oxygen was not administered but was connected in case of emergency. The scan began with a 2-minute normocapnia period during which participants breathed medical air (20.9% O_2_ balance N_2_) with a flow rate of 30 L/min. This was followed by a rapid switch to 2 min of hypercapnia where an increase in P_*ET*_CO_2_ of +7 mmHg was targeted. In total, the scan consisted of three 2-minute blocks of normocapnia and two 2-minute blocks of hypercapnia.

### Data Analysis

#### Image Preprocessing

Perfusion and BOLD weighted images were created from the first and second echo data respectively. Physiological noise correction was carried out using a modified RETROICOR technique (Glover et al., 2000) to remove cardiac and respiratory noise components from the BOLD and CBF task data. First and second harmonics of the respiratory and cardiac cycle along with the interaction term were regressed from the raw CBF signal (before tag and control subtraction) in a general linear model (GLM) framework. In addition, variability related to CO_2_, O_2_, respiration and heart rate were removed from the SRT task run (Birn et al., 2009). Surround averaging was applied to the BOLD weighted images to remove contamination from perfusion weighting (Liu and Wong, 2005). Perfusion signal modeling was carried out within FEAT (FMRI Expert Analysis Tool, FMRIB’s Software Library, RRID:SCR_002823)^1^ to model the difference between control and tag images in the timeseries.

#### Task Response Modeling

One subject was excluded from the analysis due to a low task response rate. The remaining 19 subjects’ BOLD and CBF task responses were analyzed using a GLM within FEAT with high pass filtering (cut off 80s). The voxelwise GLM was used to identify statistically significant BOLD and CBF responses across all sequence blocks. Further contrasts modeled linear response changes over time, using the task timing regressor to model the average change across the experiment as opposed to a block by block change. Cluster based thresholding was applied to define significant BOLD and CBF task responses which were threshold-adjusted using a standard voxel-level *z* score >2.3, *p* < 0.05, family-wise error corrected. A second FEAT GLM was carried out with contrasts set to calculate BOLD and CBF responses for each individual task block.

Individual subject’s functional data were registered to the high resolution T1-weighted structural image using FLIRT, FMRIB’s linear image registration tool (Jenkinson and Smith, 2001), with six degrees of freedom. The high-resolution images were then registered to the Montreal Neurological Institute (MNI) standard space with 12 degrees of freedom. FEAT contrasts were set up to investigate positive and negative task vs. rest activity. All subjects’ data were then entered into a higher-level FEAT analysis to define functional regions of interest (ROIs) for further analysis.

#### Definition of ROIs

Regions of interest were created from regions where there was a linearly decreasing component to the task-induced-signal (in both BOLD and CBF data) over sequence blocks, to investigate training related adaptation. All reported ROIs were created from the intersection between BOLD and CBF task responses unless otherwise stated. Random blocks were not included in ROI creation. These areas were then separated into anatomical regions using the Harvard-Oxford cortical and subcortical atlases within FSL. For each ROI, the parameter estimate (PE) for each stimulus block was used to calculate the percentage CBF change or BOLD response for each stimulus block.

#### Calculation of CVR and CMRO_2_

Cerebrovascular reactivity to CO_2_ was calculated according to the method described previously (Bright and Murphy, 2013), where the beta weight calculated for the CO_2_ regressor reflects the percentage BOLD or CBF signal change caused by hypercapnia and is normalized by the change in in end-tidal CO_2_ (mmHg). CVR was calculated using the voxel-averaged timeseries in each ROI. As the timing of the hemodynamic response is not uniform across the brain and there are delays in the physiological response to CO_2_, the end-tidal CO_2_ regressors were selected based on which of the 97 time shifts applied produced the best fit to the data. The delay was optimized using cross correlation between the regressor and the ROI timeseries shifted in steps of 0.1 s (Bright and Murphy, 2013). Ninety-seven time shifts were used as this was the maximum number possible given the length of the physiological recordings.

The Davis model (Davis et al., 1998) was used to calculate CMRO_2_ from normalized BOLD and CBF data in each ROI. The hypercapnia measurement was performed to estimate the scaling parameter *M* (see eq.1) which represents the estimated maximum BOLD signal response upon washout of all deoxyhaemoglobin according to the calibrated fMRI equation (Davis et al., 1998) (eq. 2). This model assumes that the targeted level of hypercapnia does not change CMRO_2_. The values for α and β must also be assumed. In the model, α represents the change in CBV as a function of CBF, the original value of this exponent as proposed by Grubb et al. (1974) was 0.38 to describe arterio-venous blood volume. More recently it has been established that the volume of the deoxyhaemoglobin compartment, venous CBV, is what is required to calculate *M*. Chen and Pike (2010) used steady-state flow and volume changes to estimate the power–law relationship between CBV and CBF and the model fit produced a coefficient of 0.18 which is comparable to simulation work (Griffeth and Buxton, 2011) and values between 0.18 and 0.23 and are more commonly used at 3T. However, being a biological parameter, actual α values are likely to vary with age, health status and under different experimental conditions.

The parameter β which equals 1.5 in the original equation is a constant representing the relationship between blood oxygenation and the BOLD signal. As relaxivity is field dependent an optimized value of 1.3 tends to be used at 3T (Mark et al., 2011; Bulte et al., 2012). Values of α = 0.20, β = 1.3 were used in this study (Bulte et al., 2012).

(1) $$M=\frac{{\text{B}}_{\text{H}}-1}{1-F_{\text{H}}^{-\left(\beta -\alpha \right)}}$$

(2) $$r\,C\,M\,R\,O_{2}\,\left(t\right)=F\,{\left(t\right)}^{1-\alpha /\beta }\,{\left(1-\frac{B\,\left(t\right)-1}{M}\right)}^{1/\beta }$$

Equations for the calculation of M (Eq. 1) and relative CMRO_2_ (*rCMRO*_2_) (Davis et al., 1998) (Eq. 2). B_H_ represents the fractional BOLD signal change during CO_2_ breathing F_*H*_ represents the change in CBF from baseline, α is the estimated ratio of fractional change in CBF to CBV and β which represents the field strength dependent relationship between blood oxygenation and the BOLD signal, *F*(*t*) = CBF at time (*t*), *B*(*t*) = BOLD at time (*t*).

#### Statistical Analysis

Statistical analyses were carried out using R (RRID:SCR_001905)^2^ and SPSS version 20.0 (IBM Corp., Armonk, NY, United States, RRID:SCR_002865). The false discovery rate was used correct for multiple corrections where such corrections were necessary; a corrected *p*-value of <0.05 was considered significant in all cases. To derive an overview of block-by-block responses, repeated measures ANOVAs with tests for sphericity were used to compare each block to a zero baseline, and to compare responses in sequence blocks 1, 2, and 6 against each other, as this is where we expected the greatest differences in task responses. Paired *t*-tests were carried out to identify significant changes between the first and second, and first and last blocks.

Repeated measures ANOVAs were used to investigate changes in the flow-metabolism coupling ratio *n*, the ratio of the fractional change in CBF relative to the fractional change in CMRO_2_ (Buxton et al., 2004), in each ROI and between blocks 1, 2, and 6.

Repeated measures ANOVAs were again conducted for behavioral data to identify significant reaction time changes from sequence block 1 vs. all subsequent sequence and random blocks. Reaction time was then used to investigate relationships between behavior and neurophysiological data. Finally, multiple linear regression analysis was used to investigate the relationships between reaction time and imaging-based responses.

Outliers in the BOLD, CBF and CMRO_2_ data were identified using Tukey’s method (Tukey, 1977) where the interquartile range is multiplied by 1.5 to define a reasonable range.

## Results

### Behavioral Responses: Performance Accuracy Across Sequence Blocks

**FIGURE 2:**
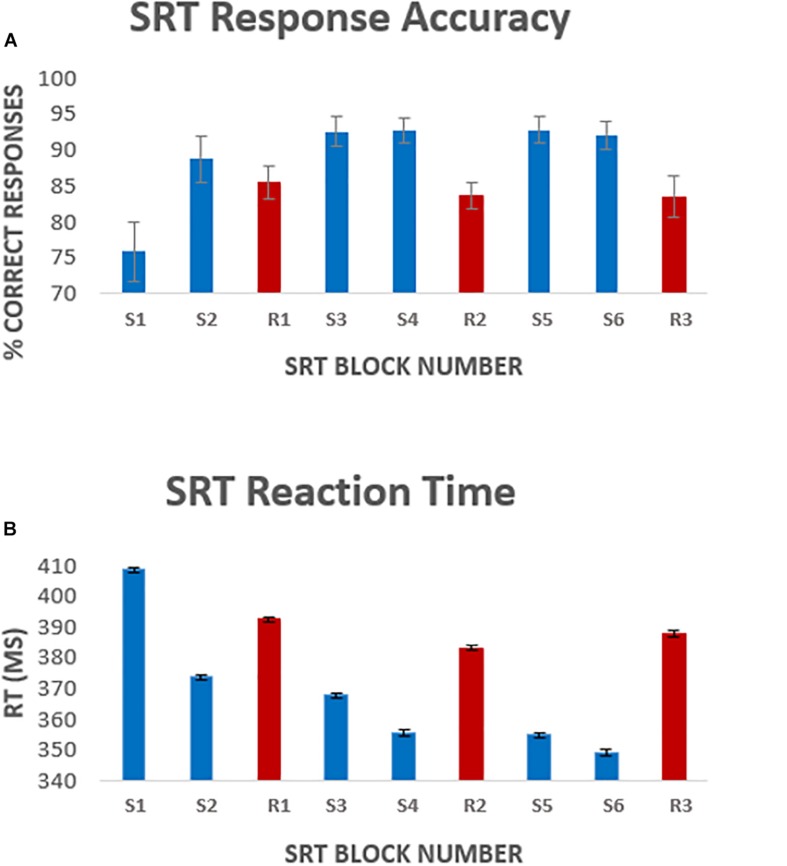
Average [mean ± standard error of the mean (SEM)] response accuracy per block **(A)**, and average response latency **(B)** per task block. Error bars represent the standard error of the mean across participants. Blocks S1–S6 represent the 6 sequence blocks, responses to these blocks were the focus of the analysis. Blocks R1, R2, and R3 (shown in red) represent pseudorandom sequence blocks.

A one-way repeated measures ANOVA was carried out to compare performance across sequence blocks. Mauchley’s test for sphericity was significant with *p* < 0.001 therefore degrees of freedom were corrected using Greenhouse-Geisser estimates of sphericity, ε = 0.441. The results showed that there was a significant effect of time on performance; response accuracy improved over time; *F*(2.2, 38.3) = 12.47, *p* < 0.001. Follow up comparisons indicated that all subsequent blocks had a significantly higher accuracy rate than block one. There was a 16.9 ± 9% accuracy improvement from sequence block S1 to sequence block S6, *p* < 0.001. Accuracy was higher in sequence blocks than random blocks, except for sequence block S1 which had the lowest performance of all blocks (Figure 2A). Random blocks R1 and R2 had significantly lower accuracy than sequence blocks 3–6. However, random block R3 scores were only significantly lower than sequence blocks S3, S4, and S6, all *p* < 0.05.

There was a significant effect of time on reaction speed; *F*(5, 85) = 298, *p* < 0.001. Follow up comparisons showed that reaction time decreased by 5.1 ± 11% from block S1 to block S6, *p* < 0.001. Random blocks had a greater response latency, again except compared to sequence block S1 (Figure 2B). Reaction time was significantly longer in random block R1 than sequence blocks S2–S6 and random block R2. Reaction time in random blocks R2 and R3 was longer than sequence blocks S3–S6, all *p* < 0.05.

### Imaging Data

**FIGURE 3:**
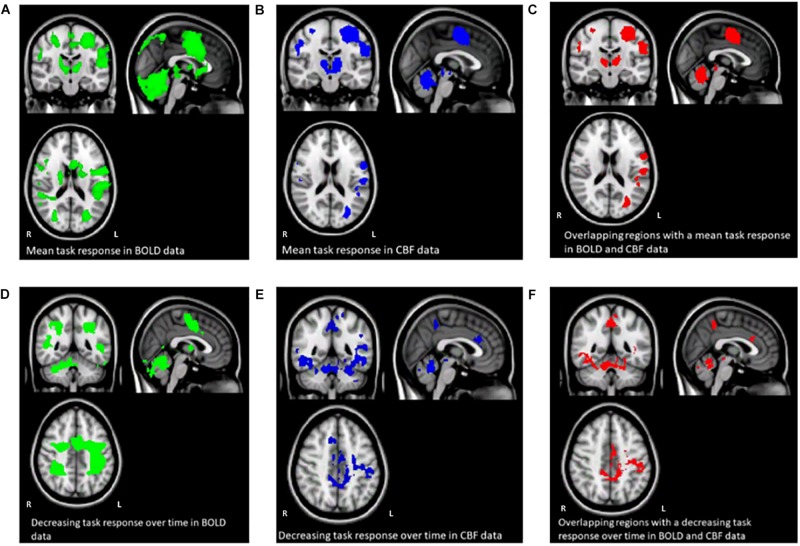
Maps **(A–C)** show the mean BOLD signal response to the task across all subjects and sequence blocks (green), the CBF response (blue), and regions with overlapping BOLD and CBF task-related signal increases from rest (red). Maps **(D–F)** show areas of BOLD signal decrease over time during the SRT task (green), decreasing CBF responses (blue) and overlapping BOLD and CBF signal decreases across the task. Random blocks were not included in this analysis.

**FIGURE 4:**
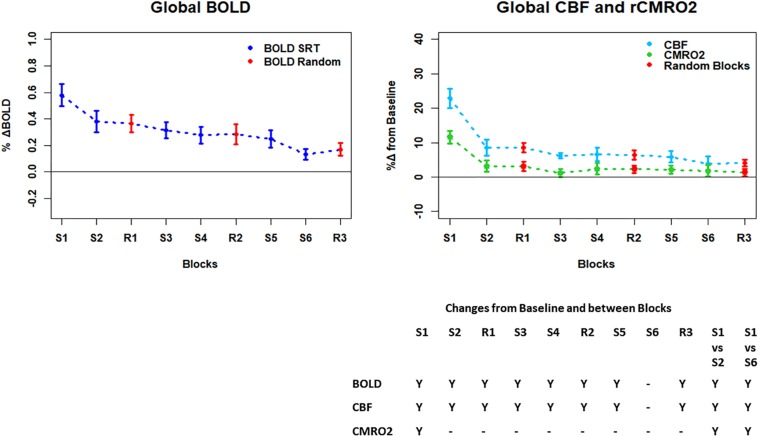
Plots showing the mean ± SEM responses for each task block in the global mean reduction ROI shown in Figure 3F. S1 = SRT sequence block 1, R1 = pseudorandom block 1. Y = Yes to indicate statistically significant (*p* < 0.05), signal changes from baseline and between first and second and first and last SRT blocks. BOLD and CBF showed significant increases from baseline across all blocks except the final sequence block S6. CMRO_2_ was significantly higher than baseline in the first sequence block only. For BOLD, CBF, and CMRO_2_, there were significant reductions in the responses between the first and second and first and last sequence blocks.

**FIGURE 5:**
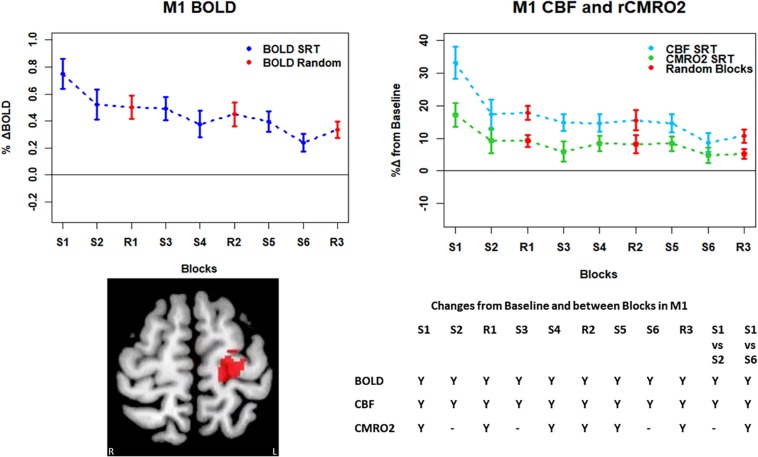
Mean ± SEM SRT task responses in M1. Plots show a mean reduction in BOLD and CBF across task blocks. S1 = SRT block 1, R1 = random block 1. Y = Yes to indicate statistically significant (*p* < 0.05), signal changes from baseline and between first and second and first and last SRT blocks. BOLD and CBF showed significant increases from baseline across all sequence and pseudorandom task blocks. There were also significant reductions in BOLD and CBF between sequence blocks S1 and S2 and S1 and S6. The CMRO_2_ was significantly elevated from baseline across all blocks except squence blocks S2 and S6. There was also a significant reduction in CMRO_2_ between S1 and S6.

**FIGURE 6:**
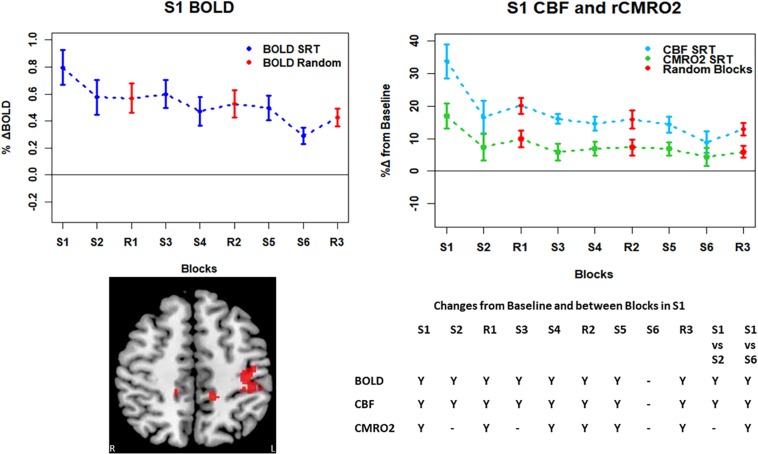
Mean ± SEM SRT task responses in S1. Plots show a mean reduction in BOLD and CBF across task blocks. S1 = SRT block 1, R1 = random block 1. Y = Yes to indicate statistically significant (*p* < 0.05), signal changes from baseline and between first and second and first and last SRT blocks. BOLD and CBF showed significant increases from baseline across all sequence and pseudorandom task blocks, except S6. There were also significant reductions in BOLD and CBF between sequence blocks S1 and S2 and S1 and S6. There was a sustained CMRO_2_ increase across all random blocks and the first, fourth and fifth sequence blocks but CMRO_2_ was not different from baseline in the second, third or final sequence blocks. As in M1, there was only a significant difference between S1 and S6 in CMRO_2_.

**FIGURE 7:**
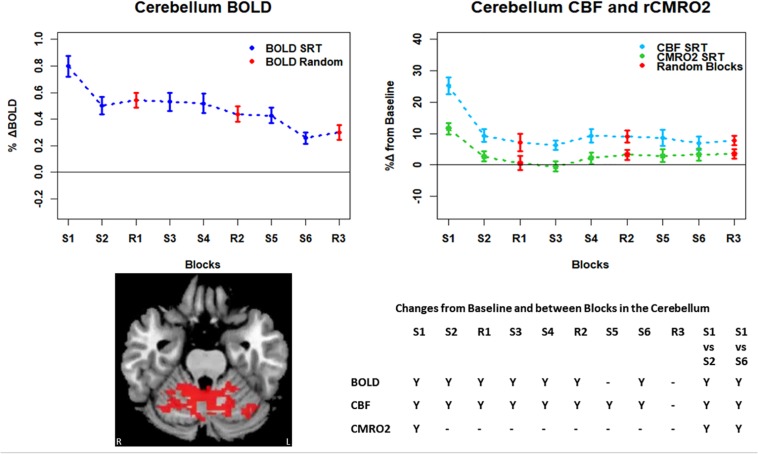
Mean ± SEM SRT task responses in the cerebellum. Plots show a mean reduction in BOLD and CBF across task blocks. S1 = SRT block 1, R1 = random block 1. Y = Yes to indicate statistically significant (*p* < 0.05), signal changes from baseline and between first and second and first and last SRT blocks. BOLD and CBF showed significant increases from baseline across all sequence and pseudorandom task blocks, except S5 and R3 for BOLD, and R3 for CBF. There were also significant reductions in BOLD and CBF between sequence blocks S1 and S2 and S1 and S6. CMRO_2_ increases from baseline were only detected in the first sequence blocks and there were significant reductions from S1 in S2, and S6.

Figures 3A–F shows the mean BOLD and CBF task positive responses, and regions where task-induced signal responses decreased on average across all subjects and task blocks as well as the conjunction between BOLD and CBF responses in each case. There were no areas of statistically significant CBF, or BOLD response increases across the task blocks; there were only decreases. Table 1 shows the group mean CVR, *M* and flow-metabolism coupling ratio values for each ROI shown in Figures 4–7. Several subjects were rejected from each ROI following the outlier removal process described in section “Definition od ROIs.”

**TABLE 1:** Mean (SEM) CVR. *M* and flow-metabolism coupling ratio (*n*) values for each ROI shown in Figures 4–7.

**ROI**	**BOLD**	**CBF**	**M%**	**Flow-Metabolism**	**Number of outliers**
	**CVR%/mmHg**	**CVR%/mmHg**		**Coupling Ratio (n)**	**removed from imaging data**
Global	0.17 (0.09)	2.4 (1)	8 (0.5)	1.45 (0.95)	1
Cerebellum	0.23 (0.04)	2.5 (0.3)	8 (1.7)	1.58 (1.02)	2
M1	0.2 (0.02)	2.3 (0.3)	13 (3.6)	1.2 (0.5)	1
S1	0.2 (0.03)	2.2 (0.3)	17 (5.6)	1.4 (0.8)	3

### BOLD, CBF, and CMRO_2_ Responses in Each Task Block

Regions of interest for further analysis were defined from the intersection between all areas showing both BOLD and CBF task response reductions (global reduction ROI, shown in Figure 3F) and subdivided using the Harvard-Oxford atlas (50% tissue probability threshold) into anatomical regions hypothesized to be central to the motor task: the pre-central gyrus (M1), the post-central gyrus (S1) and cerebellum. Figure 4 shows the group mean BOLD, CBF and CMRO_2_ responses for each task block across the global reduction ROI and Figures 5–7 show the same data for M1, S1 and the cerebellum.

Significant CMRO_2_ changes between blocks were observed in the global reduction ROI, the cerebellum and between the first and last blocks in M1 and S1. Relative CMRO_2_ significantly above baseline was observed in the first sequence block only for the global ROI (Figure 4) and the cerebellum (Figure 7). Increases from baseline were also observed in M1 and S1 (Figures 5, 6) for the first sequence block, but in addition to this, positive CMRO_2_ responses were also detected in all random blocks (R1–R3) as well as sequence blocks S4 and S5. Statistically significant BOLD and CBF increases from baseline were detected in almost all blocks in all regions, as well as reductions between the first and second and first and last blocks being observed in all regions. However, only M1 and the cerebellum showed significant BOLD and CBF responses for the final sequence block (S6).

### FMRI and Task Reaction Time

Regression analysis was conducted to determine whether reaction time was predicted by BOLD, CBF or CMRO_2_ task-related responses. For this, we focused on the first and last SRT task blocks. Multiple linear regression was carried out across subjects for each ROI with reaction time as the dependent variable, and BOLD, CBF, and CMRO_2_ as independent variables. Neither BOLD, CBF nor CMRO_2_ task responses were predictors of reaction time at the beginning or end of the task (data not shown).

## Discussion

In this study we have shown reductions in the sizes of BOLD, CBF, and CMRO_2_ responses with time in multiple brain regions recruited during performance of an SRT task. The pattern of changes was heterogeneous across regions, but the largest response amplitudes occurred in the first task block for all brain regions considered. BOLD and CBF responses were sustained (significantly raised above baseline) across nearly all blocks in the regions investigated. In the amalgamated group of regions defined by task related reductions in BOLD and CBF over time and in the cerebellum, a subset of these regions, a sustained positive CBF response was also observed in the absence of a significant positive CMRO_2_ response after the first sequence block.

The greater reaction time for random task blocks, compared to the sequence blocks, was not reflected by greater BOLD or CBF responses, and reaction time in SRT blocks did not correlate significantly with the imaging data, despite fMRI responses and reaction time both decreasing in a similar fashion across blocks. Therefore, the BOLD, CBF or CMRO_2_ responses cannot be assumed to reflect sequence specific learning directly. What we are demonstrating, is the flow and oxygenation changes occurring with repeated execution of a motor sequence, and therefore this study has important implications for the study of plasticity and higher cognitive processes such as learning. With appropriate tasks for examining the function of interest in future studies, CMRO_2_ could be calculated during early and late phases of learning as well as before and after behavioral or drug interventions to study energetic changes during learning and memory processes, and to evaluate the neural changes brought out by interventions.

### Energetic Adaptations During the SRT

Task responses and response reductions over time were identified in regions typical of a visuomotor learning task, namely M1, S1, and the cerebellum, key areas involved in motor performance. The cerebellum is critically involved in motor learning and coordination of voluntary movement. The observed decreases in the CMRO_2_ response in this area across blocks may therefore represent a shift toward automated performance of the motor task, given the sustained CBF response and CMRO_2_ near baseline levels. The sustained CBF response in the absence of significant CMRO_2_ response may suggest a preparatory or anticipatory component to energy supply. Further, the CMRO_2_ data suggests that motor adaptation occurred quickly, with greater effort required for the first sequence block and random blocks. The sustained BOLD and CBF data, in all but the final blocks may be a result of the preparatory motor response (Gjedde et al., 2004) with a smaller, or in some blocks negligible, subsequent metabolic increase.

The CMRO_2_ responses observed in M1 and S1 may be explained by ongoing recruitment of these regions for motor execution, rather than adaptation or learning. As shown in Figures 4–7, the greatest responses in BOLD, CBF, and CMRO_2_ are always observed in the first SRT block. This is likely to be a result of the initial demands of the task, focusing visual attention on the task and selecting the correct motor responses, as well as processing task information to identify the repeating sequence. In this task, subjects quickly adapted to the task demands, as evidenced by the rapid improvement in accuracy and reduction in response time. This fast adaptation may explain the sharp reduction in BOLD and CBF responses, as well as the small, and sometimes non-statistically significant CMRO_2_ increases from baseline in later blocks, as this adaptation led to a reduced neuronal demand.

While the neurobiological phenomena discussed above may explain the data, it must also be kept in mind that the CMRO_2_ measurements obtained using calibrated fMRI are prone to noise and therefore lower power than individual optimized measurements of each parameter. CMRO_2_ measured using calibrated fMRI being derived from BOLD and PASL CBF signals suffers from a lower CNR ratio than BOLD, limiting the detectability of small CMRO_2_ changes. As a result, changes from baseline may not have been detected in some instances, whereas BOLD and CBF changes, more robust over short timescales, were detected.

Signal reductions over task blocks observed in this study have been reported previously for BOLD and PET (Honda et al., 1998; Floyer-Lea and Matthews, 2004) with the largest reduction also occurring between the first and second task blocks. Fernández-Seara et al. (2009) used pulsed continuous ASL (PCASL) to investigate CBF changes during two separate 6-minute explicit learning tasks where a different pattern of sequential finger movements was trained in each task. To investigate learning-related changes, three learning phases were defined representing early to intermediate learning. CBF decreases relative to CBF measured during a control block were reported bilaterally for regions recruited during task performance, and perfusion reached levels comparable to baseline by the final task block in agreement with many ROIs in the current study. However, in contrast to the current study, where no perfusion increases over time were found, Fernández-Seara et al. (2009) found perfusion increases with task practice in somatosensory cortex, the posterior insula and putamen, cingulate cortex and left hippocampus which may be due to differences in the task design or PCASL sequence, which provides increased perfusion signal over PASL.

Although significant changes in CBF and CMRO_2_ without accompanying BOLD changes were not observed, acquiring CBF and CMRO_2_ data is still valuable as it can provide an insight into the main processes contributing to the measured BOLD signal. This is evident where BOLD and CBF remain elevated from baseline across the task despite CMRO_2_ being close to, or reduced from, baseline, as observed in the global task ROI and cerebellum, and a number of mid-task blocks in M1 and S1. In agreement with Buxton et al. (2014), the CMRO_2_ response suggests more rapid adaptation to the task than the BOLD or CBF data; it is possible that neural adaptation occurs faster than the hemodynamic response adaptation. Changes in later blocks became more variable across ROIs, but overall CMRO_2_ responses were close to baseline, in contrast to the larger responses seen in the first SRT block. This pattern may be similar to previously reported rapid adaptation followed by slower, more gradual adaptations which are likely to continue beyond the short task duration (Doyon and Benali, 2005).

### Behavioral Responses to the SRT

Brain measures did not correlate significantly with gains in performance in the form of accuracy and reaction time. Contributing to this observation may the low level of task difficulty such that ceiling effects were observed in the performance data, alongside very low variability in performance across subjects. Coupled with the high variability of the brain data, this may explain why no correlations were observed. Therefore, although motor adaptations were observed in the form of performance improvements and signal reductions, the signal changes cannot be directly interpreted as sequence specific motor learning. In the pseudorandom task blocks performance was lower, however, there was no significant difference in BOLD or CBF in these blocks compared to the SRT tasks before and after (data not shown). This suggests that the changes in BOLD, CBF and CMRO_2_ were not due to sequence specific motor learning but more to adaptation to repeated finger movements. Also, performance improvements over time can largely be attributed to sequence familiarization rather than more general motor skill improvements.

### Limitations

The principal limitation is the low CNR ratio of CMRO_2_ estimates, largely arising from the low CNR of the ASL CBF measurement. Previous work studying cerebrovascular changes during cognitive tasks has generally used much longer paradigms than the 12-minute task used here, with some exceptions (Fernández-Seara et al., 2009). Only 6 sequence blocks were included here in order to limit the duration of the scan session, meaning that SNR was relatively low for the purposes of looking at individual 45 s block activity. Additional blocks would allow “chunking” of the data into early, intermediate and late blocks to examine differences between stages of motor adaptation with increased SNR. For example, Olson et al. (2006) incorporated three 20-minute SRT blocks in their protocol which served to dilute the effects of transient anomalies in activity and build a more reliable picture of average group responses. While the 12-minute task is useful for measuring short term adaptation differences between patients and controls and to limit head motion and fatigue effects, to quantitatively assess motor adaptation or learning, longer paradigms should be used along with larger sample sizes to establish whether the variability observed in this study is due to real individual differences or simply the low CNR inherent in ASL data.

We decided to restrict our analysis to the larger regions of interest that are central to motor skills due to limited contrast-to-noise (CNR) ratio inherent in ASL, and thus CMRO_2_ estimation. The low CNR may also have affected our estimations of *M.* There is also the possibility that baseline physiology differences between brain regions and individual contributed to *M* variability, which in turn affected CMRO_2_ estimations. Future studies investigating similar processes could include more detailed measurements of baseline physiology to inform differences in CMRO_2_ responses that may not be strictly driven by the task. Recently published voxelwise *M* maps acquired using hyperoxic and hypercapnic calibration (Englund et al., 2019) found that gray matter *M* values ranged from 8.5 to 11.7%, depending on the calibration method used, in data from 10 subjects. Our estimates in the global ROI and cerebellum are in line with this, however, M1 and S1 estimates were higher at 13 and 17% respectively. However, the values of *M* were consistent across subjects and as the main function of interest is the change in task responses across blocks, the *M* constant was deemed unlikely to result in unreliable results. The limited CNR prevented more detailed investigation of flow-metabolism coupling ratio *n* and whether this changes across blocks during tasks such as the SRT. Establishing regional flow-metabolism coupling values in the healthy brain would also be useful for comparison with metabolic changes in disease.

## Conclusion

This study demonstrates the use of calibrated fMRI to detect regional BOLD, CBF, and CMRO_2_ responses to a short motor adaptation task, and the changes in the amplitude of these responses over time in a sample of healthy adults. The most interesting result of this study is the finding that in the cerebellum there was a sustained BOLD hemodynamic response in the absence of a significant CMRO_2_ increase from baseline. This suggests that the brain may continue to elevate the supply energy even after actual utilization (CMRO_2_) has reduced to near baseline levels. Therefore, relying on BOLD data alone in behavioral studies can mask the nature of underlying metabolic responses and their changes over time with repeated task performance. With refinements to the task and MR acquisition, calibrated fMRI could be used to study energetic changes during learning in the healthy brain and to investigate the vascular and metabolic changes underlying reduced cognitive and motor function and limited plasticity in aging and disease.

## Data Availability Statement

The raw data can be made available by the authors upon request.

## Ethics Statement

The study was reviewed and approved by the Cardiff University School of Psychology Research Ethics Committee and performed in accordance with the guidelines stated in the Cardiff University Research Framework (version 4.0, 2010).

## Author Contributions

CF conceived and designed the study with input from RW and VT. CF and JS coordinated the project and collected data with assistance from DH. CF analyzed the data. CF prepared the manuscript. RW provided the input and interpretation of results as well as reviewing and editing the final manuscript. All authors approved the manuscript before submission.

## Conflict of Interest

The authors declare that the research was conducted in the absence of any commercial or financial relationships that could be construed as a potential conflict of interest.

## Footnotes

1 www.fmrib.ox.ac.uk/fsl

2 https://www.R-project.org
